# Theranostics and contrast agents for magnetic resonance imaging

**DOI:** 10.1186/s40824-018-0130-1

**Published:** 2018-07-27

**Authors:** Yohan Jeong, Hee Sook Hwang, Kun Na

**Affiliations:** Department of Biotechnology, Center for Photomedicine, The Catholic University of Korea, 43 Jibong-ro, Wonmi-gu, Bucheon-si, Gyeonggi do 14662 South Korea

**Keywords:** Magnetic resonance imaging, Contrast agent, High relaxivity, Targeting

## Abstract

**Background:**

Magnetic resonance imaging is one of the diagnostic tools that uses magnetic particles as contrast agents. It is noninvasive methodology which provides excellent spatial resolution. Although magnetic resonance imaging offers great temporal and spatial resolution and rapid in vivo images acquisition, it is less sensitive than other methodologies for small tissue lesions, molecular activity or cellular activities. Thus, there is a desire to develop contrast agents with higher efficiency. Contrast agents are known to shorten both T1 and T2. Gadolinium based contrast agents are examples of T1 agents and iron oxide contrast agents are examples of T2 agents. In order to develop high relaxivity agents, gadolinium or iron oxide-based contrast agents can be synthesized via conjugation with targeting ligands or functional moiety for specific interaction and achieve accumulation of contrast agents at disease sites.

**Main body:**

This review discusses the principles of magnetic resonance imaging and recent efforts focused on specificity of contrast agents on specific organs such as liver, blood, lymph nodes, atherosclerotic plaque, and tumor. Furthermore, we will discuss the combination of theranostic such as contrast agent and drug, contrast agent and thermal therapy, contrast agent and photodynamic therapy, and neutron capture therapy, which can provide for cancer diagnosis and therapeutics.

**Conclusion:**

These applications of magnetic resonance contrast agents demonstrate the usefulness of theranostic agents for diagnosis and treatment.

## Background

Since X-rays were discovered by W.C. Roentgen, medical imaging techniques have been contributed the accurate diagnosis [1]. There are a lot of medical imaging techniques that have been developed over 100 years including magnetic resonance imaging (MRI), computed tomography (CT), gamma ray imaging, and ultrasound for accurate diagnosis (Table 1).

**Table 1:** Imaging technique for diagnosis

Imaging modality	Type of probe	Sensitivity	Spatial resolution	Advantages	Disadvantages	Reference
MRI	Gd, Mn, Ln, Iron oxide, Iron platinum	Low	25–100 μm	No radiation High resolution (soft tissue)	Slow scan High cost Noise	[109–111]
X-ray, CT	I, Au, Bi, Xe	Low	20–100 μm	Low cost High resolution (bone fractures)	Radiation	[112, 113]
Gamma ray	PET (18F, 68Ga) SPECT (99mTC, 111In, 177Lu)	High	1–2 mm	High sensitive High resolution (biological processes) Less noise	High cost Radiation	[114, 115]
Ultrasound	Microbubbles	low	50–500 μm	No radiation Fast scan Non-invasive Ease of procedure Low cost	Low resolution	[116]

Each imaging technique has its advantages and disadvantages, and some imaging techniques are more adaptable than others for specific diagnosis. After medical images have been obtained by various medical imaging tools, they are interpreted by radiologist for effective treatment and disease management. For assisting radiologists’ interpretation, contrast agents (CAs) have been developed for enhancing contrast effect of abnormalities such as cancer [2], edema [3], stroke [4], and fracture [5].

Recently, there have been growing interest in the combination of contrast and therapy. Theranostic is a new field of medicine which combines diagnosis and targeted therapy as a single agent [6]. Theranostic agents provide imaging as well as therapy at the same time. These features of theranostic agents offer synergetic advantages in comparison to traditional CAs that are used only to visualize the inside of the body. In order to increase the efficiency of the theranostic agent, it is necessary to increase the contrast effect at target site or to achieve their desired therapeutic effect. To increase the target specific imaging, various methods have been applied on theranostic agents. Targeting antibodies [7], peptides [8], aptamers [9, 10], siRNA [11], pH-sensitive polymers [12], temperature-sensitive polymers [13], catalyst-responsive polymers [14], light sensitive polymers [15], ultrasound sensitive polymers [16], and magnetic stimuli polymers have been investigated to enhance contrast effect at target sites. Moreover, to enhance the therapeutic efficacy, various treatment methods have been developed for various types of disease, such as chemotherapy, radiotherapy, nucleic acid therapy, phototherapy, and hyperthermia treatment.

MRI is a scanning technique based on the nuclear magnetic resonance and provides images of the inside of the body. MRI is a non-invasive, non-radiation, tomographic imaging modality that offers good resolution of soft tissue such as brain [17], heart [18], eyes [19], ligaments [20], and cartilage [21]. In addition, MRI provides high resolution images of blood vessels and organs. MRI technology is based on the manipulation of the inherent nuclear magnetic moment of endogenous nuclei. More than 60% of body weight is made of water, and the atoms of hydrogen consists of a nucleus with one electron going around it. In the absence of external magnetic field, spins of electrons are randomly aligned. However, when electrons are placed under a strong magnetic field, spins tend to align with or against the applied magnetic field, producing a net bulk magnetization aligned with the direction of applied magnetic field [22]. Then radio-frequency (RF) pulse is applied perpendicular to magnetic field where the RF pulse is produced by driving electrical currents through RF-transmit coils and generates a net magnetic moment of the nuclei. When RF pulse is removed, the nuclei is aligned to original magnetic field. During realignment, the nuclei is aligned parallel to magnetic field. This phenomenon is referred to as relaxation, and the nuclei loses energy by emitting their own RF signals. These signals are measured by a conductive field coil that is placed around the object being imaged. These signals are reconstructed into a 3-dimentional MR image through a computer program.

MR images are divided into T1-weighted and T2-weighted images. T1 decay is defined as the time after RF pulse needed for the longitudinal magnetization recover to 63% of ground state of main magnetic field of MRI scanner. T2 decay is defined as the time after RF pulse needed for the exponential loss of transvers magnetization decrease to 37% of excited state of applied magnetization [23]. In the presence of MR CAs, the relaxation times of surrounding water protons nearby CAs are shortened, increase the signal intensity creating a positive contrast effect [24]. CA is divided into T1-weighted CA, T2-weighted CA, and T1/T2 dual CA. T1-weighted CAs shorten T1 relaxation time to maximize its T1 contrast effect that results in a brightening of the MR image [25]. T1 CAs are usually made from lanthanide gadolinium, transition metal manganese, and dysprosium. T2-weighted CAs shorten T2 relaxation time to maximize its T2 contrast effect that results in a darkening of the MR image. T2 CAs are usually made from superparamagnetic iron oxide (SPIO) and superparamagnetic iron platinum (SPIP). Unlike other imaging tools, MR contrast enhancement is affected by various factors such as chemical exchange with water proton, tumbling time, electron spin state, distance between two dipoles, chemical shift, metal ions, and relative CA concentration on region of interest [24, 26–28]. Because of these factors, various attempts have been applied to enhance contrast effect on specific target regions and combine therapy with diagnosis. Table 2 shows the conventional CA for MR imaging for diagnosis.

**Table 2:** Conventional MR contrast agent for diagnosis

	Main material	Name of compound	Trade name	Target organ	Reference
T1	Gadolinium	gadoxetate	Primovist	Liver	[117]
		gadoterate	Dotarem, Clariscan	Brain and spine	[118]
		gadodiamide	Omniscan	Abnormal vascularity	[119]
		gadobenate	MultiHance	Liver	[120]
		gadopentetate	Magnevist	Glioma	[121]
		gadoteridol	ProHance	Brain and spine	[122]
		gadoversetamide	OptiMARK	Brain, spine, and liver	[123]
		gadobutrol	Gadovist or Gadavist	Angiography	[124]
		gadopentetic acid dimeglumine	Magnetol	Abnormal vascularity	[125]
		Albumin-binding gadolinium complexes gadofosveset	Ablavar or Vasovist	Angiography	[126]
		gadocoletic acid	gadocoletic acid	Angiography	[43]
		gadomelitol	gadomelitol	Angiography	[127]
		gadoteric acid	Clariscan	Brain and spine	[128]
T2	Iron oxide	Iron oxide	Feridex I.V	Reticuloendothelial system	[129]
		Iron oxide	Lumirem	Gastrointestinal tract	[130]
		Iron oxide	Sinerem	Lymph nodes	[131]
		Iron oxide	Resovist	Reticuloendothelial system	[132]

In this review, we will discuss highly target specific MR CAs that show therapeutic efficacy and imaging, and theranostic agents that combined with MRI agent and treatment including region specific MRI agents, drug loading MRI agents, hyperthermal MRI agents, and neutron capture MRI agents. In addition, their applications will be discussed in this review.

### Region specific MRI contrast agent

#### Liver

**Fig. 1:**
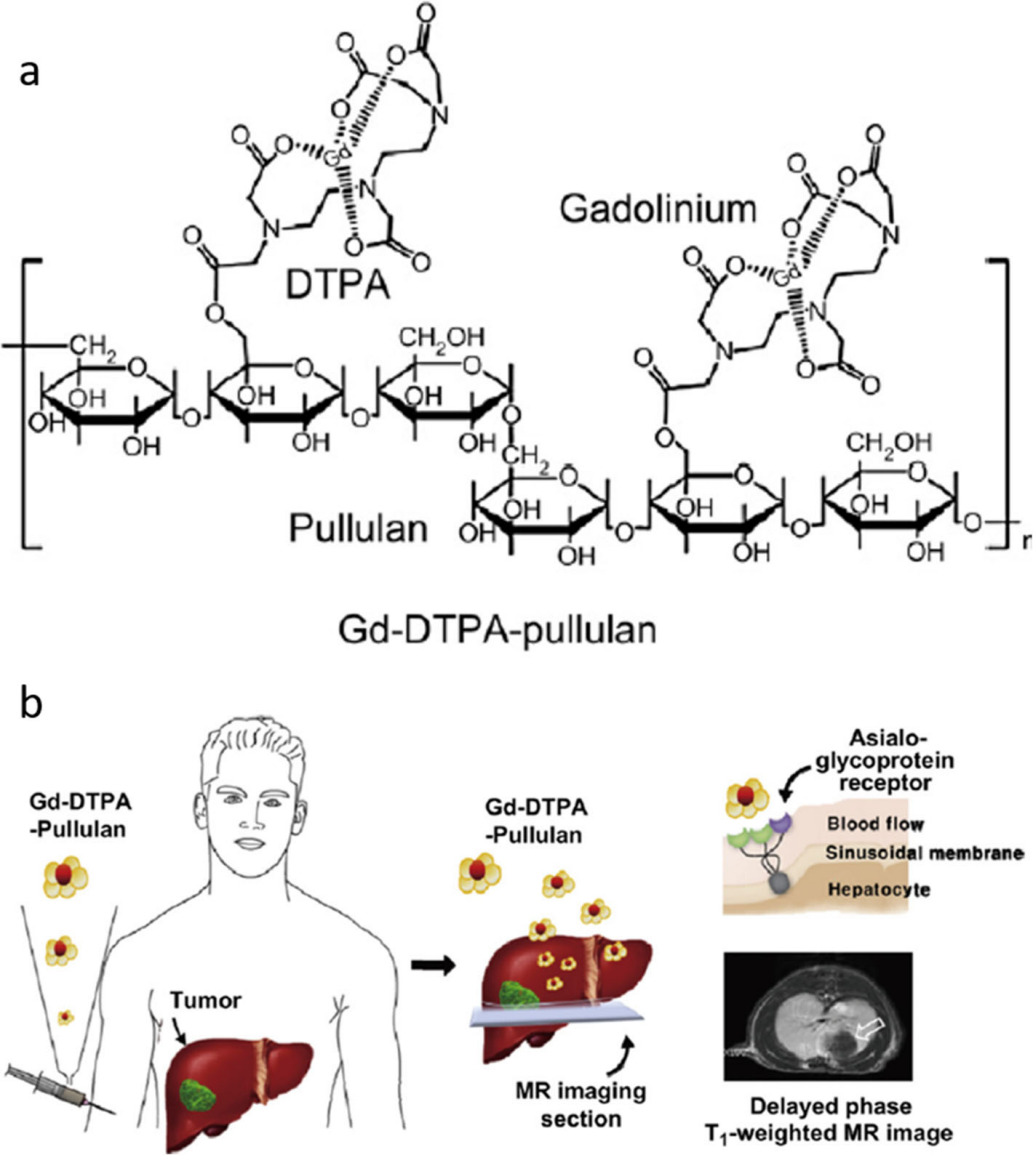
**a** A chemical structure of Gd-DTPA-Pullulan. **b** A schematic illustration of Gd-DTPA-Pullulan as a hepatocyte-specific MR agent [30]

**Fig. 2:**
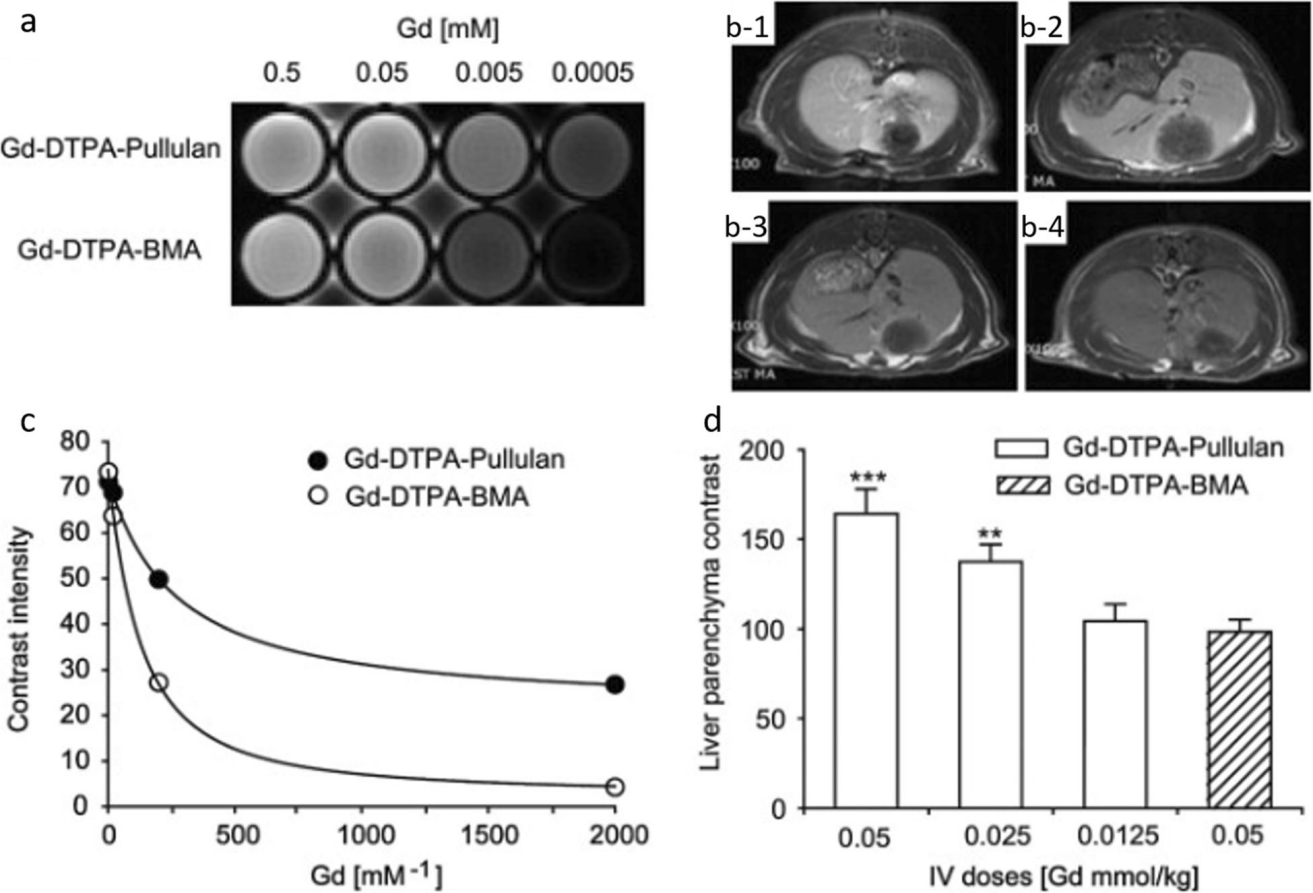
**a** T1-weighted MR images of Gd-DTPA-pullulan and Gd-DTPA-BMA. **b** Transverse T1-weighted MR images 1 h after IV injection of 0.05 (b-1), 0.025 (b-2), 0.125 mmol (b-3) Gd/kg of Gd-DTPA-pullulan and 0.05 mmol Gd/kg of Gd-DTPA-BMA (b-4). **c** In vitro contrast intensities of Gd-DTPA-Pullulan and Gd-DTPA-BMA. **d** Contrast intensities of liver parenchyma estimated using Image J software [30]

The detection of the hepatic lesion during MR imaging of the liver is clinically important for evaluation of liver metastases, since benign and malignant lesions may coexist [29]. Thus, the need of CAs that enhanced liver MR imaging is critical. According to Yim et al., pullulan-conjugated gadolinium diethylene triamine pentaacetate (Gd-DTPA-Pullulan) is designed as a hepatocyte-specific CA (Fig. 1) [30]. Gd-DTPA-Pullulan has a specific binding affinity for asialoglycoprotein receptor that are highly expressed on the membrane of hepatocytes. In vivo MR study demonstrated that intravenous administration of Gd-DTPA-Pullulan highly accumulated in the liver and showed a higher contrast intensity in liver than controls, as well as providing discriminative hepatic imaging (Fig. 2).

Mangafodipir trisodium is a manganese (Mn) chelate which is a MR CA for hepatobiliary system. It is a paramagnetic complex that is metabolized by dephosphorylation and changes to manganese dipyridoxyl monophosphate (Mn-DPMP) and manganese dipyridoxyl ethylenediamine (Mn-PLED) [31]. Mangafodipir trisodium release Mn^2+^ ions and ions are bound by alpha2-macroglobulin and transported to the liver. Mangafodipir trisodium shows greater T1 relaxivity in liver tissue than that of gadolinium because of the intracellular uptake of Mn^2+^ ions [32]. After 30 min of IV injection in rat, 13% is present in the liver. 12–20 mg manganese is presented in human body; thus, administration of mangafodipir does not show acute or subchronic toxicity [32]. The safe dosage of mangafodipir is 5 μmol kg^− 1^ body weight [33].

Gadobenate dimeglumine is an active ingredient of MultiHance which is used for IV injection for MR imaging of focal liver disease. It is an octadenate chelate of the paramagnetic ion gadolinium. Gadobenate dimeglumine distributes extracellular fluid space and selectively taken up by hepatocytes [34]. In the liver, increased intracellular viscosity within the hepatocytes allows a higher relaxivity about 20 mmol^− 1^ s^− 1^ [35, 36].

Gd-Ethoxybenzyl-DTPA (Gd-EOB-DTPA) is a hepatobiliary CA with hepatocellular uptake via the anionic-transporter protein [37]. A T1-relaxivity in water at 0.47 T is 4.9 mM^− 1^ s^− 1^, which is comparable to gadopentetate dimeglumine (3.7 mM^− 1^ s^-1)^, whereas, T1-relaxivity in human plasma is R1 8.2 mM^− 1^ s^− 1^, which is higher than gadopentetate dimeglumine (R1 5 mM^− 1^ s^− 1^). A possible reason might be due to greater degree of protein binding compare to that of gadobenate dimeglumine [38].

#### Blood-pool contrast agents (BPCA)

Natural macromolecule-derived blood-pool CAs include Gd-based MRI CAs, Gd-DTPA covalently linked to proteins such as albumin, IgG, fibrinogen, inulin. Among proteins, albumin conjugated to Gd-DTPA is the most studied and the relaxivity at 0.25 T is 14.9 mM^− 1^ s^− 1^ which is higher than the other clinically approved agents [39]. However, albumin-(Gd-DTPA)x has a limitation to the intravascular space, slow elimination, Gd association, and immunogenicity limits the use to blood-pool CA [39].

Targeted blood-pool CAs are designed to localize a specific cell or tissue which include monoclonal antibody conjugates angiogenesis biomarkers, monoclonal antibody LM609 specific to α_v_β_3_ integrin-targeted, a marker of angiogenic blood vessels. Winter et al. demonstrated that targeted nanoparticles are bound to α_v_β_3_ integrin epitopes on the aortic wall and delayed contrast enhancement of the vessel wall; thus, α_v_β_3_ integrin is successful targeted and imaging of tumors [40]. Vascular-targeted imaging using functionalized polymerized vesicles (PVs) is biotinylated anti-αvβ3 antibody (LM609) was conjugated to PVs that has specificity for endothelial cell receptors provide in vivo imaging studies in vascular associated antigens. PVs are designed to minimize reticuloendothelial system uptake and stay in the blood pool [41].

Biodegradable polydisulfide-based Gd complexes have been developed using a cleavable disulfide spacer for enhanced blood pool CAs. After MRI examination, PEG-g-poly(GdDTPA-co-l-cystine) breaks down macromolecules into smaller Gd complexes by exposure to endogenous thiols via disulfide-thiol exchange reaction to facilitate the Gd clearance [42]. In vivo MR imaging demonstrated strong contrast enhancement and large accumulation of CAs in the blood pool in mouse blood vessels, increased vascular retention, and prolonged blood pool contrast enhancement.

Protein binding blood-pool CAs include such as gadofosveset trisodium (MS-325) and gadocoletic acid trisodium (B22956) [43]. MS-325 currently in phase III clinical trials belongs to a chelated gadolinium contrast media. Breast tumor using a blood pool CA (MS-325) was compared to albumin-(Gd-DTPA)30 in rat breast tumor [44]. The intravascular binding of MS-325 to serum albumin prolonged plasma half-life, increases the T1 relaxivity, and used as a MR angiography agent.

MR monitoring of Bevacizumab anti-angiogenesis therapy has been conducted using B22956/1 in human breast cancer model as a protein-binding CA for MRI assessments of tumor microvessels and it demonstrated that contrast effect was enhanced with B22956/1 [45]. The effect of B22956/1 in anti-angiogenesis treatment was tested in rat model using an anti-vascular endothelial growth factor antibody. Data indicated that B22956/1 shows a more sensitive detection of disease progression and responses to anti-angiogenesis therapy.

### Lymph node imaging

Detection of tumor metastases in lymph nodes is critical for deciding tumor staging and planning therapies, however, small metastases in normal-sized lymph nodes are limited since current imaging techniques rely on the size and shape of the lymph node. Contrast-enhanced MR lymphography is an analysis method that provides a high contrast and resolution for the lymphatic system after administration with interstitial or intravenous applications [46].

Interstitial lymphographic CAs are highly accumulated in regional lymph nodes via fenestrated lymphatic capillaries and transport the lymph fluid to the lymph nodes. Interstitial lymphographic CAs include such as gadolinium chelates, superparamagnetic iron oxide particles (SIPO), ultrasmall superparamagnetic iron oxide particles (USPIO), liposome, micelle, and polymers. As an example of lymphographic CAs, subcutaneous injection of gadopentetate dimeglumine (Gd-DTPA) in dogs and human visualized the lymphatic pathways from the injection sites and showed enhanced accumulation in lymphatic vessels [47]. Gd-DTPA provided a selective assessment of lymph drainage from the tumor and showed the potential application against the early stage breast tumors.

### Atherosclerotic plaques

MRI is a promising method for atherosclerotic plaque imaging. Atherosclerosis is a major contributor to coronary cerebrovascular disease, myocardial infarction, and artery disease [48, 49]. There is a need for targeted and effective CAs to allow noninvasive imaging of the cholesterol-rich atherosclerotic plaques in arteries [50]. High-density lipoprotein (HDL) based CA is one of the agents that targets atherosclerotic plaques. It is a bilayer nanodisk which is composed of phospholipids and known to interacts with atherosclerotic plaques [50, 51]. Frias et al. reported that high-density lipoprotein (HDL)-like nanoparticle CA selectively targets atherosclerotic plaques [52]. In vivo MRI showed that most of the CA localized at the atherosclerotic plaque and MRI contrast intensity was maximum in plaques at 24 h post-injection. In addition, (HDL)-mimicking MRI CA using apoA-I-mimicking peptide 37pA, apoA-I mimics has been reported to show effectiveness in plaque treatment in atherosclerosis mouse model and considered as a heart disease drug [53].

Reconstituted high-density lipoprotein (rHDL) nanoparticle platform enriched with Gd-based amphiphiles are applied as a plaque-specific MR imaging CA which allows better detection of vulnerable plaques. Chen et al. reported that Gd-loaded rHDL nanoparticles are highly accumulated in atherosclerotic plaques and enhanced the vessel wall with strong MR signal intensity. In details, they developed two palmitoyl chains have been conjugated to A2, the apolipoprotein E (apoE) derived peptide, to create P2A2. P2fA2 is the lipopeptide that P2A2 is modified with carboxyfluorescein. Using the P2fA2 enriched rHDL (rHDL–P2A2) nanoparticles, they showed enhanced detection of intraplaque macrophages that are associated with plaque vulnerability [54].

### Tumor

Targeting and imaging of tumor vasculature plays a critical role to predict tumor response to therapy and to monitor of tumor angiogenesis. Thus, angiogenesis, growth, and metastasis of tumors, needs to develop accurate and non-invasive imaging CAs.

Rijpkema et al. illustrated that dynamic contrast-enhanced (DCE) MRI data is able to characterize tumors in humans [55]. CA using gadolinium (Gd) is used for the assessment of human tumors via IV bolus injection of Gd and monitored in time by T1-weighted MRI. The data provided tumor treatment response and rate of CA uptake in the tumor to evaluate treatment response and outcome. T1-weighted DCE MRI image showed that tumor was clearly distinguished from the surrounding tissues and suggested a potential of detecting metastasis.

Tumor targeting and imaging using quantum dots (QDs) [56] are also one of MR imaging CAs. For specific targeting, QDs are conjugated to an antibody for a prostate-specific membrane antigen (PSMA) for active tumor targeting. Bander et al. demonstrated that radiolabeled monoclonal antibody as a targeted prostate cancer metastasis showed enhanced accumulation and prolonged PSMA antibody at the tumor sites [57]. In addition, QDs conjugated to arginine-lysine-aspartic acid (RGD) peptides to target tumor vasculature displayed a specific affinity to angiogenic factor which is expressed in growing tumors [58].

Superparamagnetic iron oxide nanoparticles (SPION) applications in MRI provided higher contrast enhancement in MRI than conventional Gd-based CAs [59, 60]. Poly(TMSMA-r-PEGMA)@SPION which has a surface anchoring moiety and a protein-resistant moiety that is able to detect tumors in vivo using clinical MRI. SPION in the tumor area shows accumulation of iron oxide in the tumor tissue, successfully target the tumor tissue possibly via the EPR effect. When SPION administered intravenously to xenograft mice, T2-weighted MR images showed the specific accumulation within the tumor sites. After 4 h post injection, T2 signal dropped due to fast clearance, which indicates that cancer imaging can be obtained up to 4 h [60].

To overcome limitations of nonspecific CAs, targeted CAs for MRI is designed by direct conjugation of an antibody or targeting moiety to a contrast agent. For example, avidin−biotin system was coupled to a dendrimer-based macromolecular MRI CA [61]. and conjugation of avidin and biotin provides targeting of Gd ions per antibody binding [62]. These reports demonstrate that targeting molecules may increase the delivery of the CA into specific regions for effective cancer diagnosis.

**Fig. 3:**
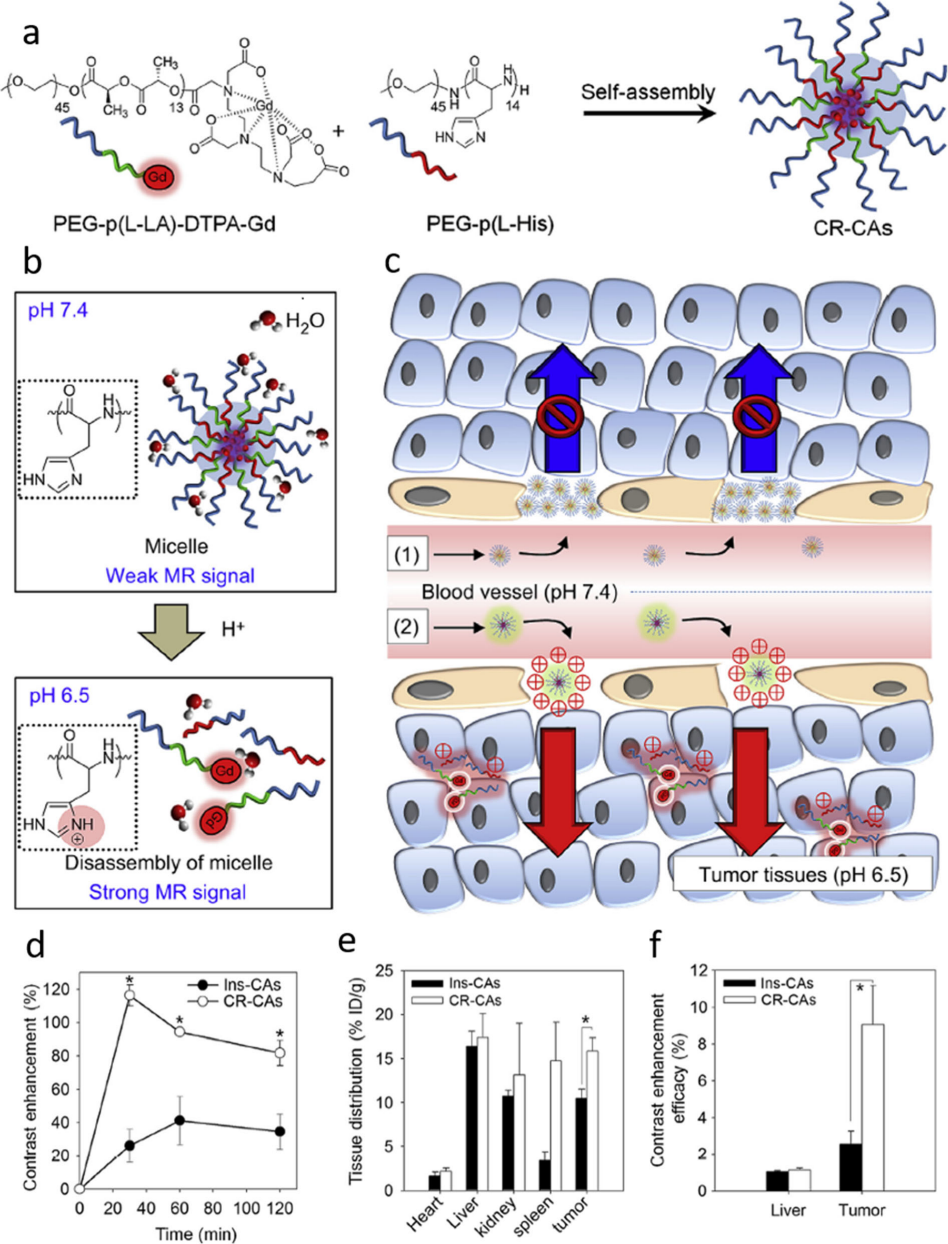
**a** Preparation of the cancer-recognizable MRI contrast agents (CR-CAs). **b** Schematic illustration of pH-dependent structural transformation and related MR signal changes in CR-CAs. **c** Schematic illustration of the tumor-accumulation behavior of conventional micelle (1) and CR-CAs (2). **d** Contrast enhancement vs. time after CR-CAs and Ins-CAs injection. **e** In vivo biodistribution of CR-CAs and Ins-CAs in CT26 bearing BALB/c mice. **f** Contrast enhancement efficacy of CR-CAs and Ins-Cas [65]

Artemov et al. developed a gadolinium-based MR CA to image the HER-2/neu Receptor [63]. The HER-2/neu receptor is a member of the epidermal growth factor which is overexpressed in breast cancers [64]. It has been reported that specific binding of avidin-Gd complexes to tumor cells using biotinylated anti-HER-2/neu monoclonal antibody (mAb) demonstrated Gd-labeled avidin was bind to the biotinylated mAb with high affinity [63]. Kim et al. also designed cancer recognizable MRI CAs (CR-CAs) using pH sensitive polymeric micelles [65]. The CR-Cas forms stable micelles at neutral pH with decreased T1 relaxivity (Fig. 3). Under tumor pH, the micelles break apart and switch to water soluble polymers with increased T1 relaxivity. Thus, the exposed Gd accumulated in the tumor tissue and showed strong enhanced T1 contrast over time.

### Combination of MR contrast agent and drug

**Fig. 4:**
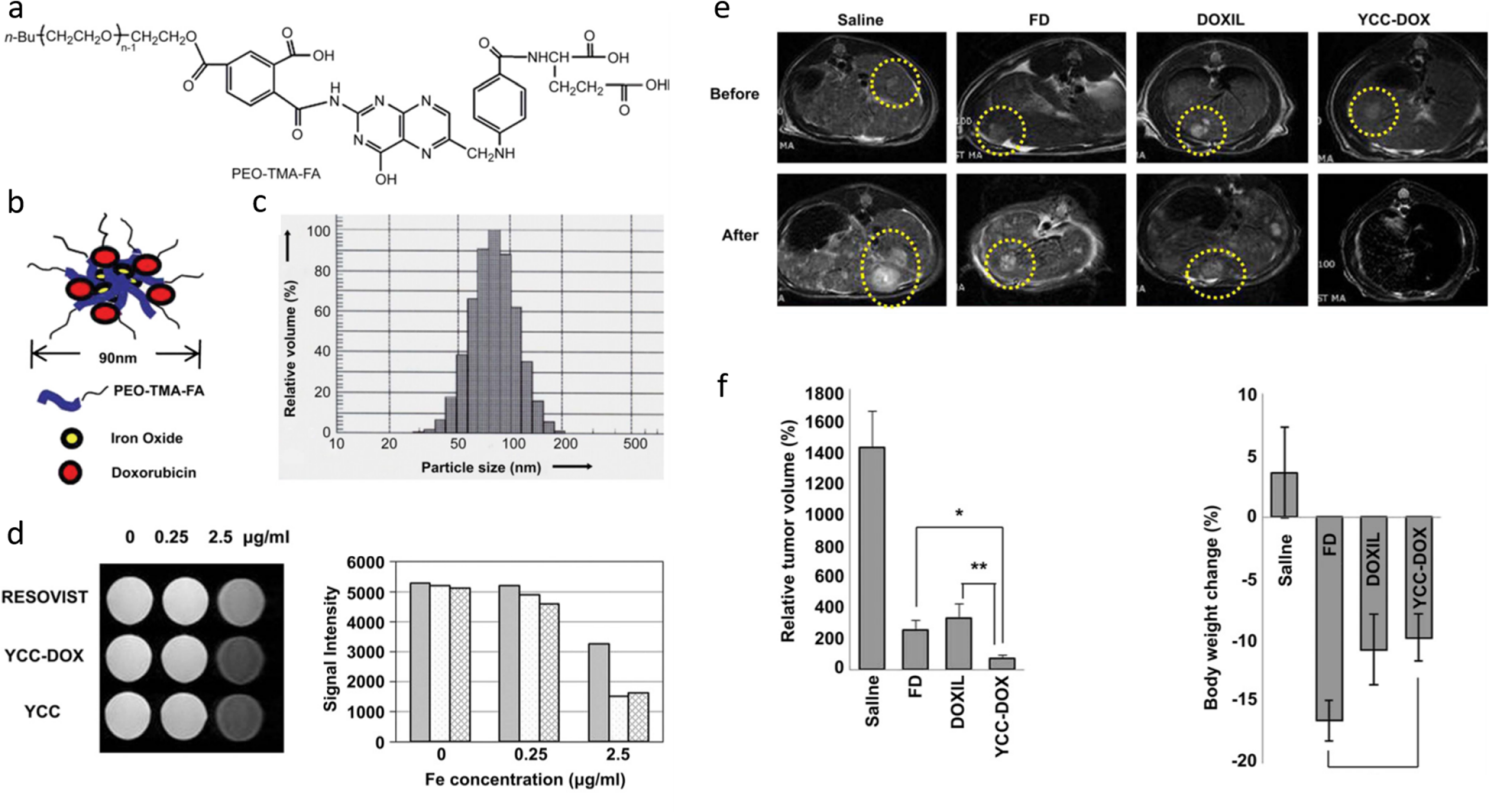
**a** Structure of PEO-TMA-FA polymer. **b** Diagram, size distribution. **c** TEM image. **d** In vitro T2-weighted MR image and the MR signal intensities of YCC, YCC-DOX, and Resovist. **e** Representative MR images. **f** Relative tumor volumes and weight changes of saline-, FD-, DOXIL, and YCC-DOX-treated rat livers [78]

Chemotherapy has long been applied in the treatment of various types of disease. However, combination of MR contrast agent and drug may overcome many limitations of conventional chemotherapy such as low targeting efficacy and side effects. To overcome the low contrast effect and low specific treatment efficacy various magnetic nanoparticles (MNP) have been developed for enhanced diagnostic. MNP is one of the nanoparticles with superparamagnetic property and used for MR contrast agent. MNPs appear to be very suitable for drug delivery and diagnosis. As well, they can be synthetized with particles of various sizes and properties in order to carry various molecules and to release in a specific environment. Recently, research has focused on the development of nanoparticle that incorporate multiple functions for multimodal imaging to enable for diagnostic at the same time. Xin Zhou et al. have recently described the properties of magnetic liposome with hydrophilic and hydrophobic drugs. Surface of magnetic liposome was coated with targeted peptide that is recognized by α_v_β_3_ integrin receptor, which is overexpressed in tumor cells. Indeed, MNPs need to be coated with polymers and peptides to stabilize them and enhance their biocompatibility. Various polymers such as poly ethylene glycol(PEG) [66], dextran [67], chitosan [68], and polyethyleneimine (PEI) [69] are used to stabilize the MNPs to make mono-dispersed particles in the solution. Also, albumin is used to avoid immunogenicity, achieve tumor accumulation, and cellular uptake of drugs for enhanced therapeutic effect and diagnosis [70]. Many chemotherapeutic drugs [71–76] and siRNA treatments [77] have already been loaded in different nanoparticles and have demonstrated a great efficacy against different types of cancers. Combination of MR CA and drug has great potential due to the numerous advantages of MNPs. For example, Maeng et al. have shown higher MR sensitivity and anticancer efficacy that MNPs loaded with doxorubicin (a potent anti-cancer agent) against liver cancer in rat and rabbit cancer models (Fig. 4) [78].

### Combination of MR contrast agent and thermal therapy

For the treatment of cancer, thermal therapies have numerous advantages. The technique is focal and repeatable with a minimally invasive application. There are 2 main types of thermal therapy: cool- or heat-based techniques [79]. Cryosurgery is based on the application of extreme cold to destroy tumors such as liver [80], lung cancers [81]. In recent years, the use of nanoparticles has led to a new technique called nanocryosurgery [82]. which was proposed to improve freezing efficiency of the conventional cryosurgery. While the use of MNPs in cryosurgery is still in its infancy, in hyperthermal treatments MNPs have been investigated for decades. For hyperthermal treatment, different techniques are available, such as lasers [83, 84], high intensity focused ultrasound [85–87], radiofrequency currents or alternating magnetic field [88, 89].

Nanoparticles have been also investigated to deliver thermal energy to tumors. The different techniques use the properties of NPs inherent to their size and composition such as optical and magnetic characteristics, thermal or electrical conductivity. For example, photothermal therapy uses laser light to heat NPs to selectively kill cells which incorporated these NPs. More recently, Gd tethered gold MNP have been shown to improve the stability and bioavailability of organic photosensitizer molecules [90]. Owing to its intrinsic high optical absorption in the near-infrared region, functionalized gold nanorod can combine both photothermal hyperthermia and imaging for optimum therapeutic efficiency [91].

The use of an external magnetic field is another technique to treat cancer with magnetic NPs. Indeed, minimally invasive magnetic heating therapy uses SPIO MNPs to generate heat (with an external alternating magnetic field) to specific tumor areas. So far, different cancer types such as brain [92], breast [93], prostate [94] and liver cancers [95–97] have been treated using this technique. The advantage of this technique is that MNPs can be injected directly into the tumor before thermotherapy and MNPs seem to remain almost completely in the tumor allowing for repeated treatments. Indeed, ultrasound mediated hyperthermal system also has been investigated for enhanced therapeutic efficacy.

### Combination of MR contrast agent and photodynamic therapy

Combination of MR contrast agent and photodynamic therapy (PDT) provides synergetic effect for treatment of cancer. PDT is a therapy consisting of visible light and photosensitizers. Photosensitizers are activated by absorption of light to generate the reactive oxygen species (ROS). ROS produced by PDT damage leads to tumor cell death and induction of antitumor immune response. In a recent research article, Jing Lin et al. have reported the current progress in the multifunctional theranostic platform based on photosensitizer-loaded gold nanoparticle(GNP). GNP encapsulate active compound for MR imaging, PDT, and photo thermal therapy using single wavelength laser irradiation [98]. Han et al. have reported the theranostic micelles based on upconversion nanoparticles for dual modality imaging and photodynamic therapy in hepatocellular carcinoma. This micelle showed noticeable antitumor efficacy compared to chemotherapy alone [99]. Skupin-Mrugalska et al. have developed the theranostic liposomes bearing gadolinium and zinc phthalocyanine as a bimodal carrier for MRI and PDT. This liposome showed enhanced contrast properties in the presence of pegylated phospholipid by increased the water proton nearby gadolinium. Indeed, cell viability of hela cell was significantly inhibited under laser exposure [100].

### Neutron capture therapy

**Fig. 5:**
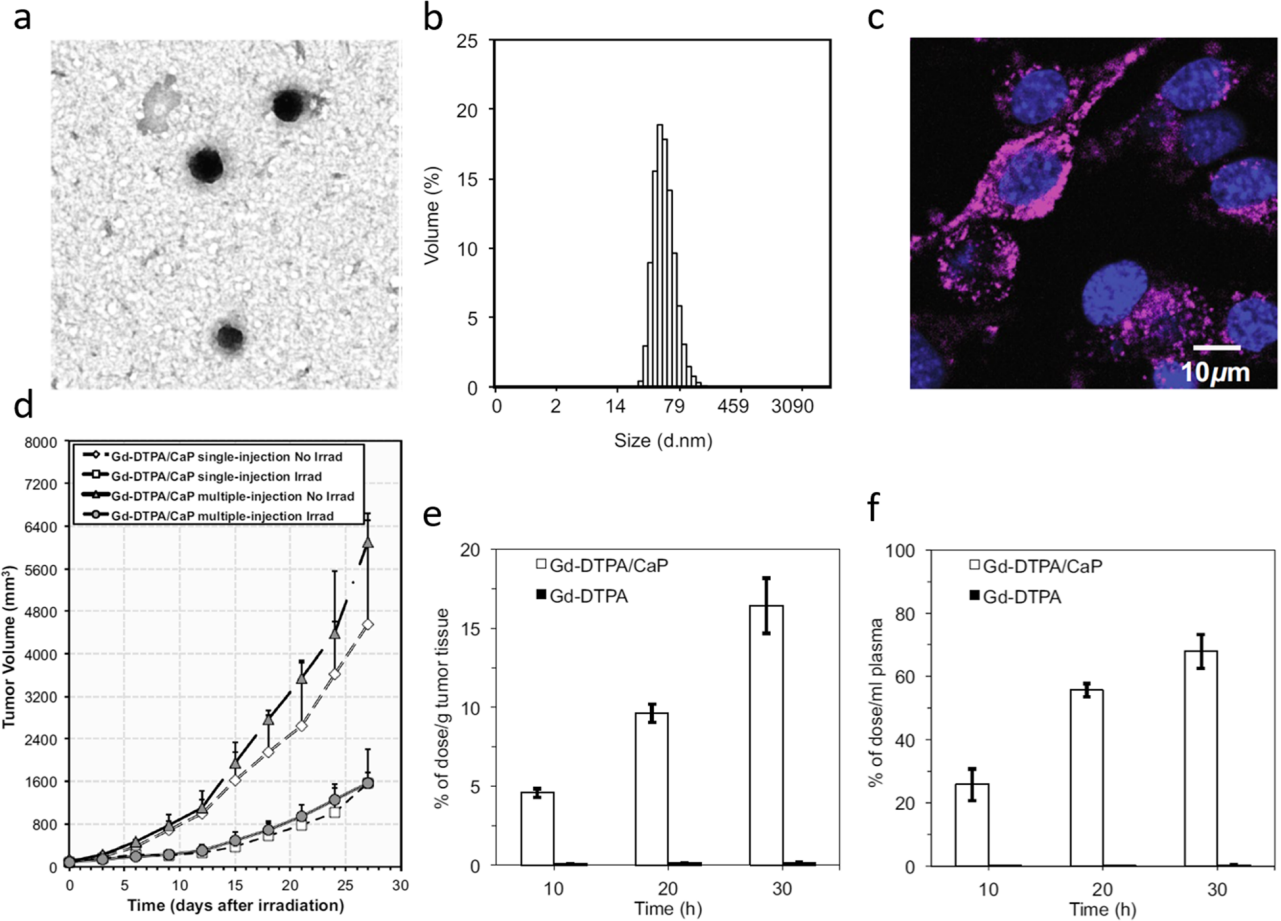
**a** TEM images of purified Gd-DTPA/CaP nanoparticles, (**b)** volume-averaged diameter distribution calculated from TEM images measured by DLS, (**c)** calcein-stained fluorescence images of Gd-DTPA/CaP nanoparticles indicating its accumulation on the surface and into cancer cells (**d**) Tumor growth suppression evaluated until 27 days after neutron irradiation for both single and multiple injections of Gd-DTPA/CaP nanoparticles. Gadolinium biodistribution for mice with multiple injections of Gd-DTPA/CaP nanoparticles, (**e**) in tumor site, (**f**) in blood plasma [128]

Neutron capture therapy is one of the treatment methods for treating early cancer, mainly using ^10^B containing molecules. ^10^B is non-reactive atom that absorb low-energy and break into ^4^He^2+^ and ^7^Li^3+^ ions, then releasing their energy at short range causing cytotoxic effect to cancer cells [101]. Unlike ^10^B, there has been interest in use of ^155^Gd and ^157^Gd as neutron capture therapy agent for several reasons. First, reaction with Gd and neutron capture induces complex inner-shell transitions that leads emission of Auger electron, γ-rays, and photon. Auger electron and γ-rays show cytotoxicity at short distances and long distances, respectively [102]. Second, Gd compounds have been used as a T1 CA for MR imaging. Because of these reasons, Gd is considered to be a suitable material for theranostic. Conventional MR CAs have been tested for neutron capture therapy. However, they showed low uptakes in tumor after intravenous injection [103]. To increase the accumulation of gadolinium in the tumor, various Gd containing polymers have been investigated. Core shell nanoparticle made of Gd and calcium phosphate(Gd-DTPA/CaP) have been synthesized. Novriana et al. have performed antitumor evaluation for single and multiple injection of Gd-DTPA/CaP nanoparticles. They could observe tumor suppression after neutron irradiation. However, there was no significant difference in antitumor efficacy between single and multiple injection (Fig. 5) [104]. In addition, various Gd containing polymers have been investigated, including Gd-loaded chitosan nanoparticles [105–107], and Gd-boron complex [108]. More recently, Dewi et al. proposed Gd-neutron capture therapy using Gd-entrapped liposome as Gd delivery agent. The concentration of Gd in tumor site was determined using ICP-MS at 2, 12, 24 h after injection. The accumulation of Gd seemed to be much higher than CA only; without entrapping it into liposome. After neutron irradiation, liposome treated group showed 4 times higher tumor suppression [107].

## Conclusion

Recently, theranostic MR CAs have been developed and various studies are conducted to enhance the contrast effect. Studies have been carried out to maximize the contrast effect by reducing the T1 relaxation time and R2 relaxivity, or to increase the water exchange rate to enhance the contrast effect. After that, the CA does not only increase the contrast effect, but also obtain a bright contrast effect in a specific condition or a specific organ, and the CA appears when the contrast is decomposed by an enzyme. A lot of researches have been investigated to synthesis materials that show treatment even with single administration. The advantages of theranostic are more convenient and efficient to treat simultaneously with enhanced contrast effect. Various methods have been tried to obtain these effects. First, the CA and the drug are sealed at the same time to be expressed at specific disease sites and simultaneously performed the contrast and treatment. As another method, there is a method of simultaneous thermotherapy with CA to generate heat and kill cancer cells. Another method is neutron capture therapy, which is a method of simultaneous treatment. Recently, variety of methods have been tried and customized theranostic materials will be developed for personalized treatment.
